# Instruments to Evaluate Food Neophobia in Children: An Integrative Review with a Systematic Approach

**DOI:** 10.3390/nu15224769

**Published:** 2023-11-13

**Authors:** Julyana Nogueira Firme, Priscila Claudino de Almeida, Emanuele Batistela dos Santos, Renata Puppin Zandonadi, António Raposo, Raquel Braz Assunção Botelho

**Affiliations:** 1 Human Nutrition Graduate Program, Nutrition Departament, University of Brasília, Brasília 70910-900, Brazil; julyanafirme@gmail.com (J.N.F.); nprialmeida@gmail.com (P.C.d.A.); emanuelebatistela.ufmt@gmail.com (E.B.d.S.); 2 Department of Food and Nutrition, Federal University of Mato Grosso, Cuiabá 78060-900, Brazil; 3 Nutrition Departament, University of Brasília, Brasília 70910-900, Brazil; renatapz@unb.br; 4 CBIOS (Research Center for Biosciences and Health Technologies), Universidade Lusófona de Humanidades e Tecnologias, Campo Grande, 376, 1749-024 Lisboa, Portugal

**Keywords:** food neophobia, children, instruments, evaluation

## Abstract

Food neophobia (FN), a frequent disorder in childhood, profoundly impacts the quality of a diet, restricting the intake of nutrients to maintain proper nutrition. Therefore, using the appropriate tools to assess FN in children to promote healthy eating habits is essential. The study aimed to develop an integrative review with a systematic approach to identify the instruments to measure FN in children and analyze their differences. The included studies (n = 17) were more concentrated in Europe, demonstrating the possible lack of dissemination of the topic at a global level. Among the 18 tools, 6 were represented by adaptations of the Food Neophobia Scale (FNS) and the Children’s Food Neophobia Scale (CFNS), and one was the CFNS itself, demonstrating the relevance of these pioneering tools. The need to meet mainly cultural and cognitive criteria led to the creation of other instruments (n = 11). A diversity of approaches concerning the respondents, age range, items, scales, and validation methods was revealed. Modifications to the tools in some nations highlighted their adaptability and effectiveness in addressing regional variations. The instruments can contribute to additional research to help us better understand the prevalence of FN in children, resulting in their health and well-being.

## 1. Introduction

Food neophobia (FN) is a frequent disorder in childhood, defined as a behavior related to the reluctance to eat new foods and accept newly introduced flavors or those with a different consistency [1]. FN is a considerable factor in determining food choices that profoundly impact the quality of a diet and plays a significant role in determining food preference [2]. All ages have an impact of FN on food preferences; although it is primarily researched in children, there is growing evidence linking fear of food to unhealthy eating habits in adults [3]. In the case of children, if they do not receive adequate treatment, the FN can follow them into adulthood. FN can be reduced in adulthood with successful management in childhood, such as using cooking-related activities or promoting flexibility and adaptation in food-related situations [4,5].

The adverse impacts of FN on children’s daily food intake [6] involve an increase in foods rich in calories but poor in nutrients [7]. Children who show neophobic behavior are more likely to be overweight because they generally eat less variety and quantity of fruit and vegetables [7]. The lack of variety in the diet, caused by FN, restricts the intake of nutrients to maintain proper nutrition in the body. When the imbalance is severe and/or long-lasting, it tends to affect various body systems, such as the nervous system, impairing the child’s cognitive and physical abilities [8]. Therefore, it is essential to choose and use the appropriate tools to assess FN [9].

The Food Neophobia Scale (FNS), created by Plinner and Hobden in 1992, was the first successful attempt to create an instrument specifically dedicated to evaluating the levels of FN in humans [10]. With ten items and evaluated by a 7-point Likert scale, the scale was validated in Canada with a sample of psychology undergraduate students [1]. The FNS has been widely used and has produced reliable results [11,12,13]; however, it consists of ten items that were created over 30 years ago [9].

Later, Pliner [14] evaluated neophobic behavior in 5-, 8-, and 11-year-old children and adjusted the FNS, developing the Food Neophobia Scale for Children (CFNS). Since 1994, the CFNS has been adjusted for many scenarios and used to assess FN levels in children [7,11,15,16]. However, other tools to measure FN in children have been created over the past decade. Some examples are the Instrument to Identify Food Neophobia in Brazilian Children by Their Caregivers [7], the Child Food Rejection Scale [17], the Trying New Foods Scale [18], and the Food Neophobia Test Tool [9]. These differ according to the respondents (child or caregiver), age group, number of items and response scale, and cultural issues.

Research into FN in children is necessary in order to understand and manage the complexity of this issue in the child development process. A previous review by Damsbo-Svendsen, Frøst, and Olsen [9] evaluated thirteen reviews of designs to assess food neophobia and willingness to try unfamiliar foods. However, the limitation was that the search was carried out in only two databases, and may have missed important information about the FN assessment tools available in other databases. Therefore, there is a need for a recent review of tools to access FN in children to provide a more complete and updated understanding of this topic.

Understanding the prevalence of FN in children is critical for promoting healthy eating habits. However, no studies have combined the available tools for assessing childhood FN. Thus, it is essential to analyze the different existing instruments, considering their particularities, because examining these differences increases the precision and comparability of research results. Moreover, understanding the characteristics of the different available instruments can help to choose the appropriate instrument according to different realities, leading to a better understanding of the impact of FN on nutrition and child development.

## 2. Materials and Methods

This is an integrative review with a systematic search. It is a thorough review of the body of literature that combines the integrative methodology of multiple sources of evidence with the systematic method of an organized and rigorous search process. To ensure openness and repeatability, this hybrid strategy involves carefully designing study questions, using well-defined inclusion and exclusion criteria, conducting exhaustive literature searches across different databases, and implementing systematic review protocols. The phases followed for the elaboration process of the integrative review were elaboration of the guiding question, search or sampling in the literature, data collection, critical analysis of the included studies, and discussion of the results.

### 2.1. Inclusion and Exclusion

Inclusion criteria were studies that included data on instruments used to identify food neophobia and its prevalence in children. It is worth mentioning that the age group varies among the studies, and all studies on food neophobia instruments for children were included independently of the age group. Exclusion criteria were: (1) letters, conferences, books, review studies, editorials, undergraduate works, and case reports; (2) studies whose target population did not involve children; and (3) studies that did not contemplate original instruments for the assessment of food neophobia in children. In the selection of studies, instruments developed for a specific population (originals) and their adaptations for children from other countries were considered as long as they met instrument validation criteria.

### 2.2. Database

Individual search strategies were developed for each database: Embase, Lilacs, Scopus, Pubmed, and Web of Science. The search for gray literature was performed on Google Scholar and ProQuest, with dissertations and theses. In addition, reference lists of studies were consulted to read the full texts of any potentially pertinent studies. The last search across all databases was performed on 24 January 2023.

### 2.3. Search Strategy

The search in each database was customized using the food neophobia and children keyword combinations and the Boolean operators OR and AND. All references were managed by Mendeley Reference Manager v2.103.0 software, and duplicate publications were excluded using Rayyan software (Qatar Computing Research Institute-QCRI; https://rayyan.ai/cite, accessed on 11 November 2023).

### 2.4. Study Selection

The process of screening the studies was performed in two phases: In Phase 1, two researchers (JNF, PCA) separately reviewed the titles and abstracts of all references detected in the databases. Only those who met the inclusion criteria were included in the next phase. In Phase 2, the same reviewers (JNF and PCA) evaluated the full texts of the articles included in Phase 1. The third reviewer (EBS) gave the final opinion in disagreement cases. The EBS researcher carefully analyzed the reference list of the selected articles. Disagreements between JNF, PCA, and EBS were resolved by expert investigators RBAB and RPZ.

### 2.5. Data Extraction

Of the selected studies, two reviewers (JNF and PCA) collected the following characteristics of the publications: research country, authors, year of publication, title, objective, type of study, sample, method used, variables, results, and conclusions. To ensure consistency between reviewers, calibration activities were performed before the review. Disagreements were resolved through discussion, and the third reviewer (EBS) decided on issues that could not be resolved by the two reviewers (JNF and PCA). Data were systematized in tables by the reviewers.

## 3. Results and Discussion

**Figure 1:**
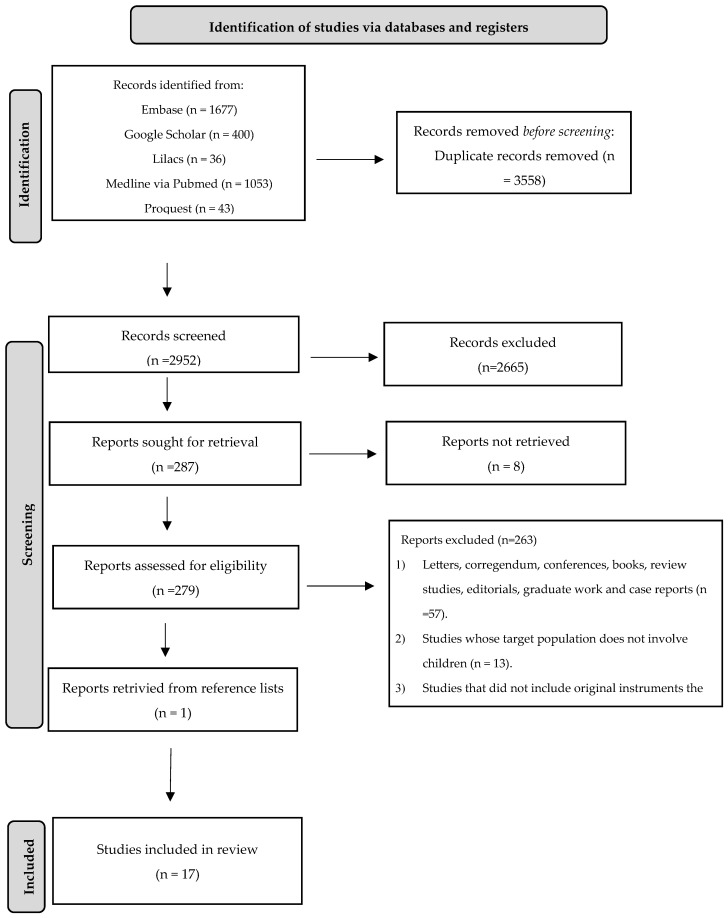
Flowchart of the integrative review with a systematic search. Adapted from PRISMA protocol [19].

The search strategies are presented in Appendix A. A total of 6510 articles were found in the databases. After excluding 3558 duplicates, 2952 articles were reviewed through their titles and abstracts. Of these, 2665 were excluded because they did not meet the eligibility criteria. Therefore, 287 studies were selected for complete reading. Of these, 17 studies were included. The selection process is described in the flowchart of the integrative review with a systematic search (Figure 1).

### 3.1. Instruments

**Figure 2:**
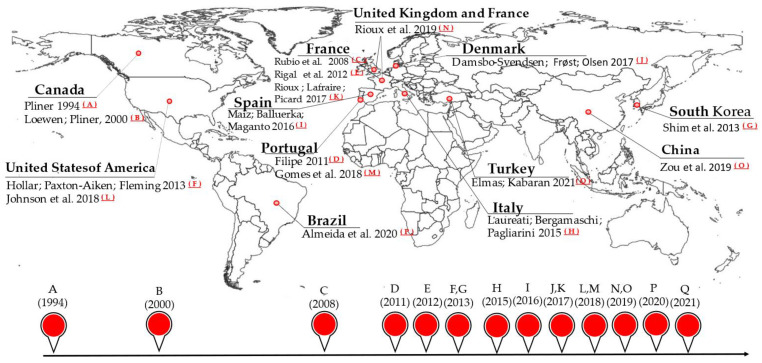
Instruments to assess food neophobia in children worldwide, 1994–2021, in chronological order.

The selected studies (n = 17) included children from 1 to 16 years old and were developed between 1994 and 2021 in the following countries: Brazil (n = 1), Canada (n = 2), China (n = 1), Denmark (n = 1), France (n = 3), Italy (n = 1), Portugal (n = 2), South Korea (n = 1), Spain (n = 1), Turkey (n = 1), United Kingdom and France (n = 1), and United States (n = 2), as shown in Figure 2.

Childhood FN has seen a remarkable transformation in understanding and treatment over the years, reflecting the global attention and concern on this issue. However, the importance of this phenomenon expanded over time, and it has caused a gradual spread of these instruments to other nations.

Figure 2 represents the availability of instruments to assess FN in several countries from 1994 to 2021. The image highlights the geographic diversity of research on FN and confirms how this phenomenon crosses cultural and geographic borders and how assessment methods have been developed throughout time precisely because of the complexity of this behavior in children. Few nations initially developed specialized tools to assess childhood FN; for example, no studies have been conducted on Oceania and Africa. Furthermore, the developed tools are concentrated in the United States, Canada, and some European countries. The illustration demonstrates the evolution of the research on FN, its global dissemination, and the continued need for updated assessment tools to understand and address this challenge.

### 3.2. Instruments and Features

The original instruments and their corresponding adaptations for children from different countries and the original instruments that were not adjusted were divided into groups to highlight the results better. The characteristics of the analyzed studies are shown in Table 1.

#### 3.2.1. Child Food Neophobia Scale (CFNS) and Its Adaptations

The Children’s Food Neophobia Scale (CFNS) comprises 10 items and were developed in 1994 by Pliner [14], and emerged from the adaptation of the Food Neophobia Scale for Adults (FNS) by Pliner and Hobden [1]. Believing that FN corresponds to a human personality trait, Pliner and Hobden [1] developed this paper-and-pencil measure of FN, which showed a high correlation with the measure of behavioral neophobia in laboratory situations. The measure presented satisfactory internal consistency (α = 0.88) and test–retest data results when applied to adults.

Previous research has shown evidence of different motivations for children (compared to adults) to reject familiar foods, motivations that also differ according to the child’s age group. Given this, Pliner proposed adapting the behavioral and paper-and-pencil measures initially intended for adults [1]. Using these new measures, the author sought to identify whether there were differences in the degree of FN concerning the age group and sex of the children, and to investigate whether child FN levels differed between foods of animal and non-animal origin and whether there were similarities between the FN of parents and children [14]. Like the FNS, the CFNS is composed of 10 items using a 7-point Likert scale, but the terms and pronouns of the FNS were modified to refer to children’s behavior reported by their caregivers. The CFNS was intended to assess the FN of Canadian children aged 5, 8, and 11 years old. The FN level is identified from the sum of the responses to each item by inverting the classifications of the neophilic items. The score can range from 10 to 70. The CFNS obtained evidence of convergent validity since the willingness to try new foods ratio and FN were correlated (r101 = 0.38, *p* < 0.001). The willingness to try new foods ratio was developed to assess behavioral neophobia.

Since its development, CFNS has been widely used worldwide [30,31,32,33]. However, in some countries, the instrument was validated for its respective population, undergoing modifications in the number of items or the response scale due to local cognitive and cultural aspects [6,9,15,20,21,22]. In the present study, adaptations aimed at children of both FNS and CFNS were identified in the continents of Europe [6,9,15,20] and Asia. Table 1 presents details of these adaptations in different countries.

Filipe [20] used the CFNS to assess FN among Portuguese children aged 5 to 6. Although the author did not make any changes to the wording or number of items of the original instrument, the author modified the response scale from 7 to 5 points on the Likert scale (“I completely agree”, “I agree”, “I neither agree nor disagree”, “Disagree”, and “Completely disagree”) and the total score varied between 10 and 50 points. In the factor analysis, the organization was maintained in one factor because the second factor included only one item, whose content was not differentiated from the first factor. The author determined the percentage of responses to the alternatives for each item (there was no need to eliminate items, as no alternative had a proportion of responses > 95%, and all alternatives were completed). They analyzed the item–total correlation (no value presented <0.20), with internal consistency (α = 0.872). Removing any item did not increase this value, and the correlation between items (items moderately correlated with each other and no value above 0.8/0.85, indicating that all items evaluated different questions).

Still in Portugal, [6] validated a Portuguese version of the CFNS for children aged 2 to 6. The authors, in addition to changing the Likert scale to 5 points (to better adapt to the characteristics of the population), excluded 2 of the 10 items of the original Canadian version due to issues related to the exploratory factor analysis, which also revealed a two-factor structure (food neophobia and food neophilia). The removed items were 5, “Ethnic food looks too weird to eat,” and 9, “I will eat almost anything”. According to the authors, the two subscales presented satisfactory internal consistency—Food Neophobia (α = 0.81; inter-item correlation mean = 0514) and Food Neophilia (α = 0.68; inter-item correlation mean = 0354)—and the subscales were significantly, moderately, and negatively correlated (rs = −0.451; *p* < 0.01). The authors described excellent test–retest reliability coefficients (rs = 0.92, *p* < 0.01 for Food Neophobia; and rs = 0.91, *p* < 0.01 for Food Neophilia). Regarding the invariance analysis, the food neophobia construct had the same structure for the two analyzed age groups. However, only partial metric invariance was found between the sexes, and concerning the convergent and discriminant validity, weak to moderate associations were found between the two subscales and other analyzed variables.

Zou [21] cross-culturally adapted the CFNS for Chinese children aged 12–36 months. The authors informed that the CFNS was translated and adapted into a Chinese version (CFNS-CN) through a forward translation, reconciliation, back-translation, expert review, and pretesting. The adaptation of this instrument, completed by caregivers, involved removing 4 of the 10 items from the original Canadian version, which were considered inappropriate by the authors for the sample’s age group. The instrument presented good internal consistency (α = 0.91) and substantial-to-good agreement between the test and retest (kappa coefficients ranged from 0.616 to 0.834).

In Italy, Laureati, Bergamaschi, and Pagliarini [15] developed and validated a self-report measure of FN for children aged 6 to 9 years old based on the adaptation of the FNS. The authors made several modifications, including the number of items, the format of the response scale, and the respondents (by the children themselves). The Italian version of the instrument (ICFNS) contains eight items (four related to neophilic attitudes and four related to neophobic attitudes). Concerns about children not understanding terms such as “ethnic” resulted in the removal of three items, replaced by one new item “I like trying new food and tastes from other countries”. The authors also changed the answer options from 7 to 5 points on the Likert scale, justifying that younger children might have difficulty discriminating between the seven options. Furthermore, they added facial figures in each answer option to help children express their opinions.

The internal consistency of the ICFNS was satisfactory (α = 0.71), and the instrument had good repeatability over the two sessions, except for younger children (6 years old). The ICFNS predicted the children’s willingness to taste and like novel food, but the ICFNS scores for the 6- and 7-year-old children were not significantly correlated with either willingness to taste or liking one of the two tested novel foods. Therefore, the authors informed that the ICFNS can be reliably used with Italian primary school children starting from eight years and most likely as early as seven years.

Aiming to develop new tools to measure FN in children aged 6 to 13 in Denmark, Damsbo-Svendsen, Frøst, and Olsen [9] presented a shortened 6-item version of the FNS [1]. In this tool, answered by the children, the exclusion of 4 items also resulted from problems with the target audience understanding terms such as “ethnic”, as well as “trust” and “particular”. The authors also changed the answer options from 7 to 5 points on the Likert scale. The results of the behavioral validation suggested that scores in 6- and 10-item versions of FNS were predictive of neophobic behavior. The authors informed that, when administered to children, the original 10-item version of FNS appeared reliable (α = 0.80) and valid (item–rest correlations, r = 0.41–0.57), but comprehension issues were evident. The shortened 6-item version of the FNS was sufficiently reliable (α = 0.72) and valid (item–rest correlations, r = 0.35–0.55). The authors found evidence for the usefulness of this shortened version to measure food neophobia without leading to comprehension issues related to items.

The most recent identified adaptation was conducted in Turkey, adapting the FNS for Turkish children aged 9 to 11 [22]. This instrument remained with 9 of the 10 items in the original version since item 10, “I like to try ethnic restaurants,” was excluded because the analysis demonstrated that it was repetitive. The response scale was modified from 7 to 5 points, using emojis to keep children’s attention. Furthermore, unlike the original version for adults, this version had children themselves as respondents. Regarding the test–retest reliability and internal consistency, the authors informed that there was no difference between the first and second test scores of all items (*p* > 0.05), and the Cronbach alpha was found to be very good for the first (α = 0.890) and for the second stage (α = 0.885).

#### 3.2.2. Food Neophobia Test Tool—FNTT

The Food Neophobia Test Tool (FNTT) was developed by Damsbo-Svendsen, Frøst, and Olsen [9] in Denmark in a study that proposed creating valid, reliable, and currently relevant tools to measure the food neophobia trait among children aged 9 to 13 years. The initial items were selected from a literature review of 13 designs created to measure food neophobia and willingness to try unfamiliar foods (134 items). The next step involved deleting items because they were not relevant to children, they were too long, or they assessed multiple topics in a single item. New items were also added by the authors, and the version at this developmental stage consisted of 19 items.

The questionnaire applied to children contained the FNTT tool and the FNS, items about willingness to try novel foods in different surroundings and a behavioral test. The questionnaire was initially developed in English, translated into Danish, and back-translated into English, so inconsistencies between words were evaluated to generate the final version in Danish. After conducting a pilot, 3 of the 19 questions of FNTT were deleted, and 3 new ones were included because the authors observed that certain aspects of food neophobia were not covered by the remaining items. Total FNTT19 scores could range from 19 to 95.

To reduce the number of items in the FNTT in order to not make it more complicated and time-consuming compared to the FNS, the authors developed three versions of the tool, containing 10, 9, and 6 items. The criteria for excluding items involved the evidence of prominent comprehension issues (in >58% of 12 classes), item–rest correlations r ≤ 0.5, a decrease in Cronbach’s α, and/or few significant item–item correlations (≥2 non-significant). The reliability of the FNTT (Cronbach’s alpha) and its validity (item–item and item–rest correlations, behavioral validation, and correlations between FNS and FNTT) were evaluated.

The authors reported that the FNTT10 and the FNTT9 were the most reliable (α = 0.91) tools, and the FNTT6 was the most valid (item–rest correlations, r = 0.67–0.80). Furthermore, they found evidence of the construct and criterion validity of the FNTT. It is important to highlight that, in the FNTT9 and FNTT10, items included led to comprehension issues in 8–75% of 12 classes, while the FNTT6 led to comprehension issues in only 8–17% of 12 classes. Therefore, the authors pondered that the latter may be a more appropriate tool, as it potentially leads to less bias than the FNTT9 and FNTT10, recommending its use in measuring food neophobia in children. In circumstances where more information is requested, they suggested the use of the FNTT9 [9].

#### 3.2.3. Food Situation Questionnaire (FSQ) and Its Adaptation

The Food Situation Questionnaire (FSQ) was developed and validated in Canada by Loewen and Pliner [23]. Before its creation, no tools measured the level of FN through children’s self-reports. Previous experiences by the authors and other groups of researchers had already demonstrated that the CFNS had some limitations due to the presence of items that addressed unusual situations and expressions not understood by children. Reports of difficulties using the 7-point Likert scale were also common. The FSQ arose from the need to address this gap, providing an easy-to-complete, self-reported measure of FN, in which the items described familiar situations and a vocabulary suitable for children.

The FSQ is an instrument comprising 10 items, which begin by describing a hypothetical situation in which new foods could be presented to children and end with a general question about the affective response, addressing different situations that may vary in terms of how to describe the food, the occasion, and who presents it. Factor analysis generated two factors that were moderately correlated (all children: r = 0.42; younger children: r = 0.39; and older children: r = 0.52) and which were retained as the following subscales: 1—Willingness to Try Novel Foods in Stimulating Circumstances (HI-STIM) represents the willingness to try new foods in highly stimulating circumstances, such as festive occasions, eating out, and accompanied by adults other than parents; and 2—Willingness to Try Novel Foods in Non-Stimulating Circumstances (LO-STIM) refers to the willingness to try new foods in non-stimulating circumstances, such as in the presence of family members, on non-festive occasions and involving “mundane” foods in meals, instead of treats.

Five facial expressions can respond to the instrument, ranging from “very sad” to “very happy”. Scores are obtained by adding the score for each subscale and the overall score of the instrument, ranging from 5 to 25 in the case of subscales and from 10 to 50 for the total scale. Higher scores indicate less neophobia as the items were described considering the willingness to try the foods. The FSQ could predict children’s real willingness to try new foods in a laboratory situation better than parents’ reports. Furthermore, it presented satisfactory reliability properties. The mean internal consistency coefficient was 0.80, and the correlation between the first and second administrations of the whole scale was 0.64.

To develop and validate a self-reported FN measurement tool for Spanish children and adolescents, Maiz, Balluerka, and Maganto [24] translated the FSQ into Spanish using the back-translation procedure. The Spanish Food Situations Questionnaire (SFSQ) was administered to a sample of 831 participants between 8 and 16 years old. The SFSQ maintained the same number of items, and factor analysis revealed a two-factor structure (as in the original instrument), but, for cross-cultural adequacy, some foods and situations described in the Spanish instrument differ from the original version (examples: cassava chips versus umami flavored chips; Halloween versus carnival; and lunch box versus afternoon snack). Furthermore, the order of the response scale was changed, starting from “very good” to “very bad”. Therefore, the higher the score, the higher the FN level, unlike the original instrument, in which the higher the score, the lower the FN level. The instrument presented satisfactory results concerning internal consistency (α = 0.77 for both the low- and high-stimulation subscales) and moderate temporal stability (Pearson correlation indices: 0.52 for the low-stimulation and 0.45 for the high-stimulation subscales). Furthermore, the Pearson correlation coefficients were used to investigate the instrument’s convergent and external validity. Total food neophobia, as measured by the Spanish version of the CFNS, had a moderate and positive correlation with the total SFSQ score (r = 0.49; *p* < 0.001) and with high-stimulation situations (r = 0.31; *p* < 0.001), and a high and positive correlation with low-stimulation situations (r = 0.57; *p* < 0.001). Concerning the external validity, the dimensions of the SFSQ were negatively correlated (in a low way) with the two subscales of the Sensation Seeking Scale.

#### 3.2.4. Questionnaire on Food Neophobia among French-Speaking Children—“QENA”

Rubio et al. [25] developed a questionnaire on food neophobia among French-speaking children (QENA). This self-reported image-based instrument has 13 items aimed at children aged 5 to 8 years old. The authors justified the need to create the instrument due to the differences in the eating habits of French children compared to those in other Western countries, for which tools such as the FSQ were created. Furthermore, they cited children’s difficulties understanding specific terms in the FSQ.

According to the authors, QENA brings together a series of unique characteristics that favor its use among French children. It uses pictures to represent foods, facilitating understanding for young children and activating brain regions that produce conceptual inferences to prove. Furthermore, the administration method (self-reported questionnaire), different consumption contexts, and the response scale (based on different types of FN) are also highlighted as strengths of the tool.

In developing the questionnaire items, the authors considered methods known to alter neophobic behavior (imitation, information, taste principle, and external stimulation). To validate the QENA, two steps were necessary. Children also completed a food task to assess the predictive validity of the questionnaire based on Pliner’s [14] methodology. In the final version of the instrument, two items use general statements about reluctance to try new foods, answered on a 4-point scale ranging from “strongly disagree” to “strongly agree”. Six items assess children’s willingness to try new foods, and five assess the FN typology.

This typology varies between without FN (referring to the child who shows a desire to try new foods), flexible FN (a child who agrees to consume the new food after trying a small piece), rigid FN (a child who consumes the new food under a pressure situation), and strong FN (a child who refuses to consume the new food). Factor analysis demonstrated a single-factor structure. The score to assess each child’s FN is obtained by averaging the item scores so that a high score indicates a strong FN. The QENA achieved satisfactory internal consistency (α = 0.84), test–retest results (r = 0.74, *p* < 0.001), and predictive validity, with scores moderately correlated with the choice of new foods (r = −0.34, *p* < 0.001) and willingness to try them (r = −0.47, *p* < 0.001). These results suggest that it is an efficient instrument for measuring NA among French children aged 5 to 8 years old.

#### 3.2.5. Children’s Eating Difficulties Questionnaire

This instrument was created by Rigal et al. [26], in a study that had the objective to validate measures of young children’s eating difficulties and maternal feeding practices in a French sample (children aged 20 to 36 months). The same study validated three other questionnaires: The Feeding Style Questionnaire, The Feeding Strategy Questionnaire, and the questionnaire relating to parental motivations when buying food for children. The study still assessed the links between maternal practices and children’s eating difficulties.

To prepare the items that made up the Children’s Eating Difficulties Questionnaire, answered by parents, and the other study questionnaires, a sample of mothers of French children aged 20 to 26 months were interviewed to investigate their children’s possible difficulties during meals and the strategies used to overcome these difficulties.

The final version of the Children’s Eating Difficulties Questionnaire, with 12 items, covers four dimensions: neophobia, pickiness, low appetite, and low enjoyment in food, but the high correlation between neophobia and pickiness and enjoyment and appetite suggested the existence of two underlying dimensions, namely, ‘‘Narrow food repertoire’’ and ‘‘Low drive-to-eat’’. The answer options range from 5 points, ‘‘very wrong’’ (1) to ‘‘very true’’ (5) for the child. The scores of six items were reversed to enable comparison.

The questionnaire was validated using a structural equation modelling (SEM) approach (with four constructs) and underwent internal consistency analysis, with a Cronbach alpha greater than 0.70 for all dimensions. The neophobia dimension, especially, presented α = 0.85.

#### 3.2.6. Fruit and Vegetable Neophobia Instrument—FVNI

The Fruit and Vegetable Neophobia Instrument (FVNI) was developed by Hollar, Paxton-Aiken, and Fleming [27] to measure students’ attitudes toward new fruits and vegetables. The study sample was students aged 8 to 10 years old, from the third to the fifth grade, collected from two evaluations of the Farm-to-School program in the United States. The FVNI, an 18-item self-report questionnaire, was adapted from the FNS. The FVNI has two subscales: a fruit subscale that asks about the child’s willingness to try new fruits in different circumstances and an analogous vegetable subscale.

Questions from Pliner and Hobden [1] were used to design the FVNI and to meet the needs of the Farm-to-School assessment [1]. Based on the FNS, two subscales, each consisting of nine items, were created in which ‘‘fruit’’ and ‘‘vegetable’’ replaced ‘‘food’’ in the original scale. The FVNI was scored on a scale of 1 to 4 for each item, with a higher score indicating greater FN.

The items dealing with foods from other countries and ‘‘constantly trying new foods’’ were not used because the children in the study sample had limited control over their exposure to culturally varied foods. Pliner’s [14] FN guided the development of additional items that asked about tasting or experiencing fruits and vegetables in various settings [14]. The study suggests that separate fruit and vegetable subscales should be employed according to the fit indices of the modified two-factor FVNI model to assess childhood neophobia.

#### 3.2.7. Assessment Tool to Evaluate the Multifaceted Characteristics of Picky Eating Habits in Children Aged 1 to 5 Years

The instrument developed by Shim et al. [28] in South Korea does not exclusively assess FN, expanding the analysis to the components of picky eating habits. However, one of these components refers to the refusal of new foods. The authors’ objective in the study that originated the tool was to evaluate the relationship between picky eating habits and the growth status of South Korean children aged 1 to 5 years old.

The authors argued that most instruments for measuring FN extracted the components through factor analysis, resulting in the union of highly related items through the subjects’ similar responses, which often had no conceptual relationship. Furthermore, they cited the existence of a study that evaluated the presence of picky eating habits in babies and young children, in which the “lack of intake” component was not evaluated and where the other components were evaluated through a question, highlighting, therefore, the need for an instrument that could solve these gaps.

The tool presents 21 items answered by parents, referring to specific eating habits reported in previous studies. Four constructs are covered: “eating a small amount” (3 items), “neophobic behavior” (2 items), “refusal of specific food groups” (9 items), and “preference for foods with specific preparation methods” (7 items). As the questions related to neophobic behavior were worded with negative words, they were scored inverted. The items referring to FN are described as “How willing is your child to enjoy new and unfamiliar food when offered?” and “How often does your child try new and unfamiliar foods at home?”. The authors use a 7-point response scale for all items. The higher the instrument score, the greater the degree of picky eating habits.

The instrument underwent a facial validity analysis, being submitted to a panel of experts in children’s eating habits, and an internal consistency assessment (α = 0.79 for questions related to the reluctance to try new foods). The authors highlighted that the tool could reflect well the multifaceted aspects of picky eating habits in children.

#### 3.2.8. Child Food Rejection Scale—CFRS

The Child Food Rejection Scale (CFRS) was developed by Rioux et al. [17] to assess FN in French children aged 2 to 7 years old. A combination of instruments was used: FNS [1], Questionnaire of Eating and Weight in Spanish Children—QENA [25], Child Eating Behavior Questionnaire—CEBQ [34], and Children’s Eating Difficulties Questionnaire—CEDQ [26]. The FN assessment instruments that existed before the creation of the CFRS were primarily developed for adults and did not sufficiently address the age range of children. As a result, the scientific literature lacked the correct assessment of FN in children.

The CFRS comprises 11 items, 6 for FN and 5 for food selectivity. The items are evaluated using a 5-point Likert scale, with coded responses ranging from 11 to 55 points. Children were presented with eight food images, four measuring selectivity and four measuring NA. The images were fixed on a plate for better understanding by the target audience.

The instrument’s two-dimensional structure, internal consistency, test–retest reliability, and convergent and discriminant validity were investigated to determine the instrument’s validity. Convergent and discriminant validity were assessed using the methodology of Pliner and Hobden [1] The results demonstrated that the CFRS scale presented good psychometric properties, is brief and straightforward, and is useful for examining FN tendencies in French children. It is essential to highlight that, in the final scale, half of the items retained for the neophobia subscale were adapted from the FNS [1], while all items retained for the selectivity subscale were explicitly created for this study.

Similar to the methodology used by Rioux et al. [17], Rioux et al. [29] validated the CFRS for the English version with caregivers of children aged 2 to 7 years old and compared the levels of selectivity and FN in children between France and the United Kingdom. English caregivers rated each item based on their child’s behavior using a 5-point Likert scale (ranging from “Strongly Disagree” to “Strongly Agree”). These responses were then quantitatively coded. For each child, three distinct scores were calculated: a FN subscore (ranging from 6 to 30), a food selectivity subscore (ranging from 5 to 25), and a total food rejection score (ranging from 11 to 55).

The authors translated and back-translated the CFRS into English before moving on to the validation and reliability assessment phases. They evaluated their construct validity, convergent validity, and reliability and conducted a confirmatory factor analysis to verify that the two-factor model found for the original CFRS by Rioux et al. [17] combined English data to assess their construct validity. The authors calculated the correlation between the CFRS and FNS points (Spearman correlation coefficient) to determine their convergent validity. Cronbach’s alpha coefficient was used to measure its consistency and reliability. The English version of the CFRS consists of 8 items, unlike the French version of the CFRS, with 11 items.

The results demonstrated that the CFRS is valid outside of France, considering that the 8-item English CFRS showed good convergent validity (CFRS scores and FNS scores highly correlated, r = 0.79, *p* < 0.001) and also good reliability (Cronbach’s alpha of 0.85). Interestingly, a reduction in the number of pertinent items is not uncommon after cross-cultural adaptation and validation [15]. These cultural variations can help guide specific actions to improve the eating habits of populations.

#### 3.2.9. Trying New Foods Scale

The Trying New Foods Scale was created by Johnson et al. [18] in the United States. This instrument assesses FN in children from the perspective of their self-competence in trying new foods. Their proposition was justified by the fact that hitherto existing measures were based, according to the authors, on the caregivers’ point of view, and the items related to feelings of fear and disgust had their origin in observations of children’s behavior or adults’ assumptions about the cause of reluctance to consume food. The authors argued that the tool could perform this measure, eliminating the need to rely on reports provided by caregivers (since it is a self-reported measure) and direct observations. The Trying New Foods Scale was developed so children can report the challenges and experiences of trying new foods.

Based on interviews with children aged 3 to 5 years old, the authors used playful resources to investigate their experience when asked to try new foods using an instrument containing a 9-item scale. The scale assesses various aspects of children’s experience, including the reasons for rejection, feelings, and consequences of trying new foods.

The description of each item is through positive and negative propositions, represented by figures that explain the content of that item (for example, “This girl likes the taste of new foods. This girl does not like the taste of new foods. Which girl is more like you?”). Each image is accompanied by a pair of circles (one large and one small) that represent the child’s frequency of identification with the given situation, such as “Always” (the large circle), “Normally” (the smaller circle) for the positive statements, and “Sometimes” (smaller circle) or “Never” (large circle) for negative statements. Thus, the answer options vary between 4 points: less neophobic/more willing to try = 4; and more neophobic/less willing to try = 1).

The principal components analysis (PCA) results demonstrated a single component with strong item–total correlations (mean ± s.d. = 3.08 ± 0.70). The instrument showed strong internal consistency (α = 0.88) and initial evidence of criterion validity, but it did not show significance in test–retest reliability (r = 0.52, *p* = 0.086). The authors attributed this fact to the small sample size involved in the test.

#### 3.2.10. Instrument to Identify Food Neophobia in Brazilian Children by Their Caregivers

The scarcity of information about FN in Brazilian children due to the lack of culturally appropriate instruments for this population led Almeida et al. [7] to develop and validate a tool capable of evaluating which types of food children are most reluctant to try. The instrument to identify FN in Brazilian children was developed from an extensive literature review, which allowed the identification and use of three tools as a basis for its preliminary version: the FN scale for adults 1992 [1], the FNTT [9]. and the FVNI [27].

After translation, these tools had their items carefully analyzed to adapt to the cultural aspects of Brazilian children. Similar items were merged, and those that did not meet the cultural issues or those not related to the age group of the sample were excluded. The researchers developed additional items. Authors describe that these additional items consider that the environment may influence eating behavior. The created items considered if the child would taste foods in different ambiances such as a friend’s house, school, or parties.

Intended to assess FN in children aged 4 to 11 years old, the instrument contains 25 items to be answered by caregivers. This has variables related to food neophobia in different environments (home, friends’ houses, school, or social events) and situations (birthday parties or friends’ meetings). Furthermore, it has three domains: general FN, FN with an emphasis on fruits, and FN focusing on vegetables. Responses vary on a 5-point scale, ranging from “Strongly Disagree” to “Strongly Agree”. The instrument’s overall score can vary between 25 and 125, so the lower the score, the greater the neophobic behavior.

The instrument presented excellent internal consistency (α = 0.958, *p* < 0.001) and reproducibility when answered by the caregiver who knows the child’s eating habits (intraclass correlation coefficient = 0.987, *p* < 0.001). Furthermore, the reproducibility analysis showed that both caregivers can also answer the instrument (intraclass correlation coefficient = 0.712, *p* = 0.003). The three domains have a similar number of items, which allows for an adequate analysis of the general score and those domains. The instrument is valid and reliable for assessing FN among Brazilian children.

### 3.3. Instrument Approach: Respondents, Age Range, Items, Scales, and Validation Methods

The discussion of the instruments used to assess FN in children revealed a diversity of approaches concerning the respondents, the studied age range, and the validation methods. The descriptors were previously studied, so the search reflected the largest number of studies with children as the target audience.

Most of the instruments (n = 14, 78%) were built to assess FN exclusively [6,7,9,14,15,18,20,21,22,23,24,25,27]. However, it is important to highlight that there is variability in how FN is measured through these different instruments. An example of these differences is that some tools have subscales specific for fruits and vegetables [7,27], differing from others like CFNS, and adaptations [14], which evaluate the general FN.

Besides that, four tools did not evaluate FN exclusively. The Children’s Eating Difficulties Questionnaire [26] involves, in addition to FN, the assessment of other possible difficulties during meals (pickiness, low appetite, and low enjoyment of food). The instrument from Shim et al. [28] evaluates, besides FN, three other dimensions: eating a small amount, refusal of specific food groups, and preference for foods with specific preparation methods. The instruments of Rioux et al. [17,29] evaluate FN and pickiness. These instruments were included because they evaluated, although not exclusively, the FN.

It is crucial to distinguish “picky eating habits”, “avoidant restrictive food intake disorder (ARFID)”, and “food neophobia” when discussing children’s eating habits [26]. A child’s selective preferences for particular foods, frequently motivated by flavor, texture, or familiarity, are considered picky eating. It is a typical stage of childhood development that most kids outgrow later. Contrarily, FN extends beyond basic fussiness [13]. It is defined by a dislike of tasting new or strange foods, frequently accompanied by apprehension or dread of unusual tastes or components. According to the Diagnostic and Statistical Manual, 5th Edition (DSM-5), ARFID is a more serious eating disorder characterized by significant dietary restrictions that can negatively impact health and development [35]. It is a disturbed pattern of feeding or eating that needs one of these characteristics to be diagnosed: failure to achieve growth in children, significant nutrition deficiency, dependence on tube feeding, or interference with an individual’s psychosocial functioning. The FN can be more enduring and hinder a child’s openness to new foods, which may impact their dietary diversity and nutritional intake [7]. Some instruments assess FN and picky eating, probably because FN is one constituent part of picky eating [36]. Recognizing and effectively resolving feeding issues in children requires understanding these variances.

Respondent-related questions are important because parents play a crucial role in feeding their children, but evaluating FN from the perspective of children has been the justification for the development of some instruments in recent years [9,15,18,25]. Relying solely on parents’ reports of their child’s FN underestimates the child’s role in the process [15,36]. Even so, half of the analyzed instruments chose to evaluate the perspective of parents or caregivers, which reflects the perception of only one side. This can be explained, in part, by the fact that some of these tools are old, such as CFNS (which, despite being widely used, is approximately 30 years old), and others are products of its adaptations [6,14,20]. Furthermore, some instruments were applied to babies, and very young children [21,26] and, in some cases, the online method was used to obtain answers, situations that would make it difficult for the children themselves to fill out the instruments [7]. It should be noted that, when creating questions for children, some attention should be taken, such as changing items to describe situations that children are likely to be familiar with, using age-appropriate language, and providing a clear response format [15,23]. In addition, there are concerns about how different groups and cultures might perceive and understand specific FN statements [15,37].

As a result of the search and data analysis, the children’s age ranged between 1 and 16 years old since one of the instruments was built to evaluate FN in children and adolescents. In this sense, the study that included adolescents was selected since the authors also evaluated children’s FN. Concerning the age group, we observed an emphasis on instruments that investigated children of preschool and school ages (3 to 10 years old), predominantly among children aged 5 [6,7,14,17,18,20,25,28,29] and 9 [7,9,15,22,23,24,27]. This concentration can be attributed to the perception that these age groups are more susceptible to the understanding and cognitive manifestation of FN, given their stage of development and food exploration [2].

In the review, 70% of the studies evaluated the effect of age on FN. Among the included studies, 67% observed no difference concerning the age groups assessed [6,9,14,17,18,20,28,29]. Zou [21] and Rigal [26] evaluated children aged 1 to 3 years and observed that children from 2 years were more neophobic. For older age groups, Loewen and Pliner [23] described greater neophobia among children aged 7 to 9 than those aged 10 to 12, and Elmas and Kabaran [22] identified that children aged 10 were less neophobic than those aged 9 and 11. Even though some studies have presented differences in age groups, as the present review did not aim to evaluate the prevalence of FN among children, it is impossible to affirm that the prevalence of FN varies according to age because most studies showed equal behavior regardless of age group.

Some studies (n = 4; 23%) presented the prevalence of FN varying from 21% to 56% [15,20,21,28]. However, it is impossible to compare the prevalence since the authors used different instruments and forms of classification.

Sex differences were also explored in 70% of the studies. Among these, 83% did not find different levels of FN between sexes [6,9,14,17,20,21,22,23,29]. Among the studies that found some difference, FN was higher among boys aged 1 to 3 years [26] and higher among girls aged 3 to 5 years [18]. Not all studies explicitly included data that would allow an exploration of FN prevalence based on sex or age.

This lack of pattern regarding sex or age with higher degrees of FN represents an important consideration for the conclusions of our study [23]. There was no standard classification among the studies, whether in percentage or degree of FN. We suggest it is an important gap in research and providing valuable information about possible variations in FN between different demographic groups would offer a favorable avenue for future investigations on the topic.

The acceptance or rejection of food can be strongly influenced by the culture and context in which the child grows up [8]. Some foods may be considered taboo in certain cultures, while others may be celebrated. It is essential to consider the cultural context when creating an assessment tool and adapt it, if necessary, to reflect cultural specificities.

Authors like Maiz et al. [24] modified items of the instrument to make them more appropriate to Spanish culture. The foods listed in items 7 and 10 of the original FSQ are cassava and chayote, respectively; they are translated as “cassava” and “chayote” in Spanish. Because the purpose of these items is to introduce new and unfamiliar foods, and because some Spanish-speaking children may be familiar with these two foods, the “umami flavor” is replaced with “cassava” (cassava) and “chucander” (an Indian flavor vegetable) to “chayote”. The review revealed a variety of options for the most popular rating scales, including 4-, 5-, and 7-point rating scales.

In the Brazilian instrument, categories that were not representative of the Brazilian scenario or had no influence on assessing children’s FN were eliminated. There were no synonyms because Brazil is a country with a wide variety of cuisines and strong cultural influences, so items that mentioned ethnic foods or restaurants, for example, were excluded [7].

The study by Rubio et al. [25] emphasized the influence of cultural factors on food selection and highlighted differences in rules, consumption conditions, beliefs, meal preparation, and meal preferences between cultures. The instrument included items that described various contexts of food consumption, aiming to integrate the context in which children can find new foods and increase the instrument’s validity.

The most common scale was the 5-point scale (67%) [6,7,9,17,20,22,23,24,26,29], followed equally by the 4-point [18,25,27] and 7-point scales [14,18,21] with 16.5% each. Some scales also had facial expressions to facilitate comprehension by the children (28%) [15,18,22,23,24]. Regarding the number of items to measure FN, there was a notable variation, with a predominance of scales with ten items, totaling 25% [9,14,20,23,24]. Other instruments present few items, with the predominance of six items of FN assessment (20%) [9,17,21], and others, such as Brazil, use more extensive instruments, with 25 items [7]. This highlights the need to balance the breadth of assessment with the practicality of use while considering each research situation’s unique characteristics.

All instruments included in the present study showed evidence of validity and reliability. The most common validation steps in the instruments included construct and convergent validation, but other approaches were described as criterion, external, content, discriminant, predictive, and facial validation. Regarding reliability, the most evaluated properties were internal consistency and reproducibility, usually temporal stability.

Both validity and reliability are considered important factors to guarantee the quality of measurement instruments. Therefore, their rigorous evaluation is necessary [38,39]. Validity concerns the instrument measuring precisely what it purports to measure [40]. Construct validity assesses the degree to which an instrument can measure a concept that cannot be measured directly, the construct. Predictive validity (the ability of the instrument to predict an evaluated criterion) and concurrent validity (where scores of the measure under evaluation are correlated with the scores of another measure that evaluates the same construct) are categories of criterion validity. All evidence of validity leads to evidence of construct validity [38].

Classical test theory emphasizes the importance of reliability in measurement, asserting that any measurement result comprises both the “true” score and measurement error. Achieving a perfect score requires eliminating measurement errors, making instrument development crucial [38]. Test–retest reliability and internal consistency are key aspects of reliability assessment, evaluating item equivalence and interrater reliability [41,42].

Higher reliability coefficients (ranging from 0.00 to 1.00) signify more excellent reliability. Internal consistency, often assessed with Cronbach’s alpha, gauges item comparability and accuracy, with increased items improving measurement precision. Employing multiple items enhances measurement reliability and accuracy [38].

It is important to highlight that, despite all tools evaluating FN and most of them being developed based on the same previous tool, they differ among the number of items and scales, and some use different domains and types of items. In this sense, studies that used different tools cannot be compared since they evaluate FN in different ways.

Finally, it is essential to emphasize that this review has some limitations, including language barriers, as studies written in languages other than English were translated on virtual platforms, which may have led to the loss of some information. Furthermore, the focus of the study was to present the tools available for assessing FN in children, not including a set of studies that used these tools and their respective results. Future research could focus on gathering evidence on the results of applying these tools to different populations. As strengths, the study provides the first comprehensive and critical view of the tools used to measure children’s dietary FN, highlighting their strengths and contributions to understanding this issue. Future research can benefit from reviews such as this one by exploring the causes of childhood FN, improving the assessment tools already available to deal with it more effectively, and expanding the foundation for building future instruments, especially in countries that do not have this type of study.

## 4. Conclusions

This study presented a complete overview of the tools used to measure children’s FN by an integrative review with a systematic approach. The geographic distribution of these studies, more concentrated in Europe, demonstrated the possible lack of dissemination of the topic globally, making it challenging to identify the prevalence of FN in children in countries where validated tools are unavailable.

Among the 18 tools found in this study, six were represented by adaptations of FNS and CFNS [1,15], demonstrating the relevance of this pioneering tool in detecting FN. However, there is a need to make more current instruments available, capable of being answered by children, involving appropriate language and experiences common to this audience. Instruments that consider this group’s specificities include different age groups (from babies to older children), considering the cultural characteristics specific to each country. It is essential to highlight that cultural issues must be considered when producing an instrument to assess FN. Modifications made in the tools in many nations highlight their adaptability and effectiveness in addressing regional variations in cognition and culture.

The preponderance of measures reported by caregivers highlights the importance of parents and other caregivers in this situation. Nevertheless, it is also important to emphasize the value of considering children’s views. We can understand more about FN if we consider age-related differences, as well as the wide range of rating scales and items of the instruments.

The review also highlighted the value of using standardized tests to identify children’s FN. Even with the effort made to detect only validated instruments in the literature, it is noteworthy that it is impossible to list the best or most appropriate instrument to measure FN, because this choice will depend on specific conditions, such as information relating mainly to the age group to be studied, the country, and the individual who will respond to the instrument (child/caregiver).

Considering the study’s objective of identifying instruments to measure FN in children and analyzing their differences, the importance of considering cultural influences in the development and adaptation of such assessment tools should be considered. The impact of culture on the acceptance and rejection of foods is evident, as different societies may have different attitudes towards different foods. This requires careful consideration of the cultural context when developing instruments to assess food consumption, especially FN. In summary, this study highlights the importance of incorporating cultural adaptations in developing assessment instruments to ensure their relevance and effectiveness in diverse cultural contexts.

Childhood FN is significantly complex, needing special attention and care for a thorough and accurate assessment. Thus, using validation approaches ensures the quality of instruments to obtain diagnostic measures that support the treatment. Healthcare professionals, especially nutritionists, must keep up with the most recent assessment techniques as understanding the subject improves the design of effective feeding patterns and support systems for kids with FN. The studied instruments can contribute to additional research to help better understand and address the prevalence of FN in children, resulting in their health and well-being.

## Figures and Tables

**Table 1:** Main descriptive characteristics and results from the included instrument (n = 18).

Author; (Year); Country	Tool	Items	Validation	Likert Scale	Respondent	Age Group
Pliner [14] (1994) Canada	CFNS (Child Food Neophobia Scale)	10 items	Convergent validity (behavioral measure of neophobia)	7 points	Caregiver	5, 8, 11 years
Filipe [20] (2011) Portugal	CFNS (Portuguese version of Child’s Food Neophobia Scale)	10 items	Analysis of psychometric properties: Correlation analysis between items Determination of the percentage of response to the alternatives for each item Factor analysis Internal consistency Item–total correlation	5 points	Caregiver	5–6 years
Gomes et al.; [6] (2018) Portugal	CFNS (Portuguese version Child’s Food Neophobia Scale)	8 items	Construct validity: factor analysis Convergent and discriminant validity Forward-backward translation process Internal consistency Invariance analysis Test–retest reliability	5 points	Caregiver	2–6 years
Zou et al.; [21] (2019) China	CFNS (Chinese version of the Child Food Neophobia Scale)	6 items	Forward–backward translation Factor analysis Internal consistency Test–retest reliability	7 points	Caregiver	1–3 years
Laureati; Bergamaschi; Pagliarini; [15] (2015) Italy	ICFNS (Italian Child Food Neophobia Scale)	8 items	Predictive validity Internal consistency Test–retest reliability	5 points + figures with facial expressions	Child	6–9 years
Damsbo-Svendsen; Frøst; Olsen; [9] (2017) Denmark	FNTT (Food Neophobia Test Tool)	3 versions: 10 items, 9 items, 6 items	Behavioral validation test Correlations between FNS and FNTT Forward–backward translation process Internal consistency Item–item and item–rest correlations Behavioral validation	5 points 5 points	Child Child	9–13 years
	Shortened 6-item version of the FNS	6 items	Internal consistency Test–retest reliability			9–13 years
Elmas; Kabaran; [22] (2021) Turkey	FNS (Turkish version of the Food Neophobia Scale FNS)	9 items	Construct validity (factor analysis) Content validity Forward–backward translation process Internal consistency Test–retest reliability	5 points + figures with facial expressions	Child	9–11 years
Loewen; Pliner; [23] (2000) Canada	FSQ (The Food Situations Questionnaire)	10 items	Convergent validity (correlation between behavior and FSQ scores) Factor analysis Internal consistency Test–retest reliability	5 points + figures with facial expressions	Child	7–12 years
Maiz; Balluerka; Maganto; [24] (2016) Spain	SFSQ (Spanish Food Situations Questionnaire)	10 items	Convergent validity Dimensionality External validity Factor analysis Forward–backward translation process Internal consistency Temporal stability	5 points + figures with facial expressions	Child	8–16 years
Rubio et al.; [25] (2008) France	QENA (Questionnaire on Food Neophobia among French-speaking Children)	13 itens	Factor analysis Internal consistency Predictive validity (food task) Test–retest reliability	4 points	Child	5–8 years
Rigal et al.; [26] (2012) France	Children’s Eating Difficulties Questionnaire	12 items (3 for neophobia)	Factor analysis (structural equation modeling) Internal consistency	5 points	Caregiver	1.6–3 years
Hollar; Paxton-Aiken; Fleming; [27] (2013) United States	FVNI (Fruit and Vegetable Neophobia Instrument)	18 items	Construct (convergent) Validation—factor analysis	4 points	Child	8–10 years
Shim et al.; [28] (2013) South Korea	An assessment tool to evaluate the multifaceted characteristics of picky eating habits in children aged 1 to 5 years	21 items (2 for food neophobia)	Facial validity Internal consistency	7 points	Caregiver	1–5 years
Rioux; Lafraire; Picard; [17] (2017) France	CFRS (Child Food Rejection Scale)	11 items (6 for neophobia)	Convergent and discriminant validity Internal consistency Test–retest	5 points	Caregiver	2–7 years
Rioux et al.; [29] (2019) United Kingdom and France	CFRS (English version of The Child Food Rejection Scale)	8 items (Neophobia subscale: N1 N2 N6 N7 N10)	Construct validity Convergent validity Internal consistency	5 points	Caregiver	2–7 years
Jonhson et al.; [18] (2018) United States	The Trying New Foods Scale	9 items	Component analysis Criterion validity Internal consistency Test–retest reliability	4 points + figures with facial expressions	Child	3–5 years
Almeida et al.; [7] (2020) Brazil	Instrument to Identify Food Neophobia in Brazilian Children by Their Caregivers by their caregivers	25 items	Content validity (panel of experts) Internal consistency Semantic evaluation (panel of experts) Test–retest and interobserver reliability	5 points	Caregiver	4–11 years

## Data Availability

No data availability.

## Appendix A

**Table A1:** Databases and terms used to search reference instruments used in the world to identify the prevalence of FN in children.

Database	Search (24 January 2023)
MEDLINE via Pubmed	(“Avoidant Restrictive Food Intake Disorder”[MeSH Terms] OR “Avoidant Restrictive Food Intake Disorder”[Title/Abstract] OR “food neophobia”[Title/Abstract] OR “choosy eating”[Title/Abstract] OR “food refusal”[Title/Abstract] OR “food rejection”[Title/Abstract] OR “food aversion”[Title/Abstract] OR “feeding neophobia”[Title/Abstract] OR “picky eating”[Title/Abstract] OR “picky eaters”[Title/Abstract]) AND (“child”[MeSH Terms] OR “child”[Title/Abstract] OR “children”[Title/Abstract] OR “infant”[Title/Abstract] OR “child preschool”[Title/Abstract] OR “schoolchildren”[Title/Abstract] OR “child nutrition”[Title/Abstract] OR “child feeding”[Title/Abstract] OR “parent”[Title/Abstract] OR “parents”[Title/Abstract] OR “guardian”[Title/Abstract] OR “guardians”[Title/Abstract] OR “caregiver”[Title/Abstract] OR “caregivers”[Title/Abstract] OR “mother”[Title/Abstract] OR “mothers”[Title/Abstract] OR “father”[Title/Abstract] OR “fathers”[Title/Abstract] OR “son”[Title/Abstract] OR “sons”[Title/Abstract] OR “daughter”[Title/Abstract] OR “daughters”[Title/Abstract] OR “sibling”[Title/Abstract] OR “siblings”[Title/Abstract] OR “family”[Title/Abstract] OR “families”[Title/Abstract])
Embase	‘(‘avoidant restrictive food intake disorder’/exp OR ‘avoidant restrictive food intake disorder’ OR ‘food neophobia’:ti,ab,kw OR ‘choosy eating’:ti,ab,kw OR ‘food refusal’:ti,ab,kw OR ‘food rejection’:ti,ab,kw OR ‘food aversion’:ti,ab,kw OR ‘feeding neophobia’:ti,ab,kw OR ‘picky eating’:ti,ab,kw OR ‘picky eaters’:ti,ab,kw) AND (‘child’ OR ‘child’/exp OR child OR children:ti,ab,kw OR infant:ti,ab,kw OR ‘child preschool’:ti,ab,kw OR schoolchildren:ti,ab,kw OR ‘child nutrition’:ti,ab,kw OR ‘child feeding’:ti,ab,kw OR parent:ti,ab,kw OR parents:ti,ab,kw OR guardian:ti,ab,kw OR guardians:ti,ab,kw OR caregiver:ti,ab,kw OR caregivers:ti,ab,kw OR mother:ti,ab,kw OR mothers:ti,ab,kw OR father:ti,ab,kw OR fathers:ti,ab,kw OR son:ti,ab,kw OR sons:ti,ab,kw OR daughter:ti,ab,kw OR daughters:ti,ab,kw OR sibling:ti,ab,kw OR siblings:ti,ab,kw OR family:ti,ab,kw OR families:ti,ab,kw)
Web of Science	TS = (“School Feeding” OR “Nutrition Programs and Policies” OR “School Meal” OR “School Meals” OR “School Meal Quality” OR “School Lunch” OR “School Lunches” OR “School Food Service” OR “School Food Services” OR “Brazilian National School Feeding Program” OR “National School Food Program” OR “School Feeding Program” OR “School Feeding Programs” OR “School Feeding Programmes” OR “School Nutrition” OR “School canteens” OR “School canteen”) AND TS = (“Sustainable development” OR “Waste management” OR “Sustainable” OR “Sustainability” OR “Environmental Sustainability” OR “Economic Sustainability” OR “Social Sustainability”)
Scopus	(TS = (“Avoidant Restrictive Food Intake Disorder”) OR TS = (“food neophobia”) OR TS = (“choosy eating”) OR TS = (“food refusal”) OR TS = (“food rejection”) OR TS = (“food aversion”) OR TS = (“feeding neophobia”) OR TS = (“picky eating”) OR TS = (“picky eaters”)) AND (TS = (child) OR TS = (children) OR TS = (infant) OR TS = (“child preschool”) OR TS = (schoolchildren) OR TS = (“child nutrition”) OR TS = (“child feeding”) OR TS = (parent) OR TS = (parents) OR TS = (guardian) OR TS = (guardians) OR TS = (caregiver) OR TS = (caregivers) OR TS = (mother) OR TS = (mothers) OR TS = (father) OR TS = (fathers) OR TS = (son) OR TS = (sons) OR TS = (daughter) OR TS = (daughters) OR TS = (sibling) OR TS = (siblings) OR TS = (family) OR TS = (families))
Lilacs	‘((“Avoidant Restrictive Food Intake Disorder”) OR (“food neophobia”) OR (“choosy eating”) OR (“food refusal”) OR (“food rejection”) OR (“food aversion”) OR (“feeding neophobia”) OR (“picky eating”) OR (“picky eaters”) OR (“transtorno da evitação ou restrição da ingestão de alimentos”) OR (“neofobia alimentar”) OR (“Trastorno de la Ingesta Alimentaria Evitativa/Restrictiva”) OR (“F03.400.157”)) AND ((child) OR (Niño) OR (children) OR (infant) OR (“child preschool”) OR (schoolchildren) OR (“child nutrition”) OR (“child feeding”) OR (parent) OR (parents) OR (guardian) OR (guardians) OR (caregiver) OR (caregivers) OR (mother) OR (mothers) OR (father) OR (fathers) OR (son) OR (sons) OR (daughter) OR (daughters) OR (sibling) OR (siblings) OR (family) OR (families) OR (criança) OR (crianças) OR (“M01.060.406”) OR (“pré-escolar”) OR (“pré-escolares”) OR (escolar) OR (escolares))
